# Effects of distractors on upright balance performance in school-aged children with attention deficit hyperactivity disorder, preliminary study^☆^^☆☆^

**DOI:** 10.1016/j.bjorl.2016.10.007

**Published:** 2016-11-17

**Authors:** Fatma Esen Aydinli, Tuna Çak, Meltem Çiğdem Kirazli, Betül Çiçek Çinar, Alev Pektaş, Ebru Kültür Çengel, Songül Aksoy

**Affiliations:** a Hacettepe University, Faculty of Health Sciences, Speech and Language Department, Ankara, Turkey; b Hacettepe University, Faculty of Medicine, Child and Adolescent Mental Health Department, Ankara, Turkey; c Hacettepe University, Faculty of Health Sciences, Audiology Department, Ankara, Turkey; d Hacettepe University Hospital, Ear Nose and Throat Department, Audiology and Speech Pathology Unit, Ankara, Turkey

**Keywords:** Attention deficit hyperactivity disorder, Balance, Distractor, Sensory organization test, Transtorno de déficit de atenção e hiperatividade, Equilíbrio, Distração, Teste de organização sensorial

## Abstract

**Introduction:**

Attention deficit hyperactivity disorder is a common impairing neuropsychiatric disorder with onset in early childhood. Almost half of the children with attention deficit hyperactivity disorder also experience a variety of motor-related dysfunctions ranging from fine/gross motor control problems to difficulties in maintaining balance.

**Objectives:**

The main purpose of this study was to investigate the effects of distractors two different auditory distractors namely, relaxing music and white noise on upright balance performance in children with attention deficit hyperactivity disorder.

**Methods:**

We compared upright balance performance and the involvement of different sensory systems in the presence of auditory distractors between school-aged children with attention deficit hyperactivity disorder (*n* = 26) and typically developing controls (*n* = 20). Neurocom SMART Balance Master Dynamic Posturography device was used for the sensory organization test. Sensory organization test was repeated three times for each participant in three different test environments.

**Results:**

The balance scores in the silence environment were lower in the attention deficit hyperactivity disorder group but the differences were not statistically significant. In addition to lower balance scores the visual and vestibular ratios were also lower. Auditory distractors affected the general balance performance positively for both groups. More challenging conditions, using an unstable platform with distorted somatosensory signals were more affected. Relaxing music was more effective in the control group, and white noise was more effective in the attention deficit hyperactivity disorder group and the positive effects of white noise became more apparent in challenging conditions.

**Conclusion:**

To the best of our knowledge, this is the first study evaluating balance performance in children with attention deficit hyperactivity disorder under the effects of auditory distractors. Although more studies are needed, our results indicate that auditory distractors may have enhancing effects on upright balance performance in children with attention deficit hyperactivity disorder.

## Resumo

**Introdução:**

O transtorno do déficit de atenção e hiperatividade é um distúrbio neuropsiquiátrico comum que causa comprometimentos, com início na primeira infância. Quase metade das crianças com transtorno do déficit de atenção e hiperatividade também experimentam uma variedade de distúrbios relacionados às habilidades motoras, desde problemas de controle de habilidades motoras finas/grossas até dificuldades na manutenção do equilíbrio.

**Objetivos:**

O principal objetivo deste estudo foi investigar os efeitos de distrações, especificamente duas distrações auditivas diferentes, música relaxante e ruído branco, sobre o desempenho do equilíbrio vertical em crianças com transtorno de déficit de atenção e hiperatividade.

**Método:**

Comparamos o desempenho do equilíbrio vertical e o envolvimento de diferentes sistemas sensoriais na presença de distração auditiva entre crianças em idade escolar com transtorno do déficit de atenção e hiperatividade (n = 26) e controles com desenvolvimento típico (n = 20). O dispositivo Neurocom SMART Balance Master Dynamic Posturography foi utilizado para o teste de organização sensorial. O teste de organização sensorial foi repetido três vezes para cada participante em três ambientes de teste diferentes.

**Resultados:**

Os escores de equilíbrio no ambiente em silêncio foram menores no grupo com transtorno do déficit de atenção e hiperatividade, mas as diferenças não foram estatisticamente significativas. Além dos escores de equilíbrio mais baixos, as razões vestibulares e visuais também foram menores. As distrações auditivas afetaram positivamente o desempenho do equilíbrio geral para ambos os grupos. Condições mais desafiadoras, usando uma plataforma instável com sinais somatossensoriais distorcidos foram mais afetados. Música relaxante foi mais eficaz no grupo de controle, e ruído branco foi mais eficaz no grupo de hiperatividade com déficit de atenção e os efeitos positivos do ruído branco se tornaram mais evidentes em condições desafiadoras.

**Conclusão:**

Que seja de nosso conhecimento, esse é o primeiro estudo que avalia o desempenho do equilíbrio em crianças com transtorno do déficit de atenção e hiperatividade sob os efeitos de distrações auditivas. Embora mais estudos sejam necessários, os nossos resultados indicam que as distrações auditivas podem ter efeitos de aumento no desempenho do equilíbrio vertical em crianças com transtorno de déficit de atenção e hiperatividade.

## Introduction

Attention deficit hyperactivity disorder (ADHD) is a common impairing neuropsychiatric disorder with onset in early childhood. Children with ADHD show developmentally inappropriate levels of inattentive and/or hyperactive impulsive behaviors in multiple settings.^1^ Epidemiologic studies indicate that ADHD is prevalent throughout the world, with a general consensus that 7–9% of youths have the disorder.^2^

Almost half of the children with ADHD also experience a variety of motor-related dysfunctions ranging from fine/gross motor control problems to difficulties in maintaining balance.^3^, ^4^, ^5^, ^6^, ^7^, ^8^, ^9^, ^10^ Studies examining underlying processes show that motor impairment in ADHD is characterized by timing, coordination, and force deficits, all of which are associated with cerebellar dysfunction.^11^ ADHD symptomatology is suggested to be related with fronto-striato–cerebellar circuit dysfunctions and converging data has revealed volume reduction of the cerebellar vermis in children with ADHD.^12^, ^13^, ^14^, ^15^ Balance control requires the integration of somatosensory, vestibular, and visual sensory information and cerebellar integrity is believed to be the key process.^16^ What's more balance control also requires attention and central information processing and therefore is strongly related to the cognitive process^17^, ^18^, ^19^, ^20^, ^21^, ^22^, ^23^, ^24^, ^25^ rather than being a subcortical, pure reflex mechanism.^17^, ^18^, ^21^, ^22^ Studies in ADHD populations may provide valuable information regarding balance because of the accompanying attention, inhibition and executive function deficits. A few studies examined balance in children with ADHD and mostly concluded disturbed balance performance.^18^, ^23^, ^26^, ^27^, ^28^, ^29^, ^30^ Most studies focused on upright balance performance^26^, ^28^, ^29^ and a couple investigated the effects of different cognitive tasks on balance performance.^18^, ^23^ In a study, researchers found greater postural sway while participants were performing auditory–memory-demanding cognitive tasks.^23^ In a study, researchers examined the effect of methylphenidate on standing balance performance under three different test conditions: standing upright; performing a memory attention-demanding task; and listening to relaxing music.^18^ The results revealed that methylphenidate significantly improved postural stability while performing a dual task and listening to relaxing music. However, the study did not include healthy controls or different distractors.

There are two main ways to detect balance performance: center of pressure displacement measurements and the Sensory Organization Test (SOT). The SOT is used to evaluate the contributions of different sensory systems to standing balance control. The test is a common research tool in the evaluation of sensory organization of balance control and has been previously used by other researchers in studying balance performance in children.^28^

In summary, motor and balance impairments can be observed in children with ADHD sharing a common neurobiological basis with core ADHD symptoms. Balance control requires attention, and distractors that affect attention may also affect balance performance. Some studies have shown the effects of distractors on cognitive skills in children with ADHD^31^, ^32^; however, no study has investigated the effects of distractors on balance performance. To the best of our knowledge, no study has evaluated balance performance in children with ADHD using distractors, such as background music and white noise. The aim of this study is (1) to compare upright balance performance in school-aged children with ADHD and typically developing controls, (2) to investigate the effects of relaxing music and white noise as distractors and (3) evaluate the involvement of different sensory systems during performance in the presence of distractors.

## Methods

### Participants

In the ADHD group, children who had undergone first-time psychiatric admission and assessment were recruited from the Child and Adolescent Psychiatry Outpatient Clinic. The inclusion criteria were as follows: (1) a formal diagnosis of ADHD confirmed by two different child and adolescent psychiatrists with at least ten years of experience with ADHD according to the Diagnostic and Statistical Manual of Mental Disorders (DSM-IV-TR) and confirmed by the Schedule for Affective Disorders and Schizophrenia for School-Age Children-Present and Lifetime Version (K-SADS-PL), (2) age between 7 and 12 years, (3) normal vision with or without glasses, and (4) normal hearing status. Children were excluded from the study if they had any of the following: (1) history of a chronic neurological condition or movement disorders, (2) total IQ score below 80 on the Wechsler Intelligence Scale for Children-Revised (WISC-R), (3) significant musculoskeletal or cardiopulmonary conditions that may influence balance performance, (4) any kind of current psychotropic medication, and (5) diagnosis of a psychotic disorder, autism spectrum disorder, or developmental coordination disorder (DCD) according to the DSM-IV-TR criteria. To warrant a diagnosis of DCD, the child had to demonstrate motor coordination substantially below the expected of the child's age, which interfered with activities of daily living and academic performance. The children in the control group were matched by age and sex and were recruited from the community; they had to fulfill the same inclusion and exclusion criteria set above, except that the first inclusion criteria was set as follows: (1) no formal diagnosis of any psychiatric disorder made confirmed by two experienced a child and adolescent psychiatrists according to the DSM-IV-TR criteria and confirmed by the K-SADS-PL.

### Measures

Schedule for Affective Disorders and Schizophrenia for School-Age Children-Present and Lifetime Version (K-SADS-PL) is a semi-structured diagnostic interview designed to assess current and past episodes of psychopathology in children and adolescents according to the DSM-III-R and DSM-IV criteria.^33^ K-SADS-PL is administered by interviewing the parent(s) and the child, after which, summary ratings are provided that also include all sources of information. Gökler et al. showed the validity and reliability of K-SADS-PL for Turkish children and adolescents, and the kappa values for various disorders ranging between 0.458 and 0.875.^34^

Wechsler Intelligence Scale for Children-Revised (WISC-R), is an individually administered intelligence test that includes six verbal (General Information, Similarities, Arithmetic, Judgment, Vocabulary, and Digit Span) and six performance (Picture Completion, Picture Arrangement, Block Design, Object Assembly, Digit Symbol, and Labyrinths) subscales. Five verbal (General Information, Similarities, Arithmetic, Judgment, and Digit Span) and five nonverbal (Picture Completion, Picture Arrangement, Block Design, Object Assembly, and Digit Symbol) subscales of WISC-R were used in this study. Savaşır and Şahin showed the validity and reliability of WISC-R for Turkish children and adolescents.^35^

The Conners’ Parent Rating Scale (CPRS-48) is one of the most used behavioral scales in clinical and research settings for children suffering from neurodevelopmental disorders, particularly for children with ADHD. CPRS-48 consists of 48 items on a four-point likert scale indicating the severity of a particular behavior and is used in the evaluation of problem behaviors related to ADHD by obtaining reports from primary caregivers.^36^ The CPRS-48 Turkish version is recognized as a valid and reliable instrument in screening ADHD symptoms in both clinical and community settings in the Turkish population.^37^

The Sensory Organization Test (SOT) quantifies the efficacies of three sensory systems (visual, vestibular, and somatosensorial) in obtaining upright balance control. SOT evaluates balance performance under six conditions with gradually increasing difficulty. Somatosensorial, visual, or sensory information are distorted through calibrated “sway referencing” of the support surface and/or visual surround. These conditions are condition C1: eyes open, stable platform; C2: eyes closed, stable platform; C3: visual disorientation (sway referenced vision), stable platform; C4: eyes open, unstable platform; C5: eyes closed, unstable platform; and C6: visual disorientation, unstable platform. “Stable platform” refers to conditions in which the dynamic platform is maintained in a static stable position. “Unstable platform” refers to conditions in which the Equitest platform is referenced to the sway of the subject. The platform responded to changes in weight transfers and shifts in the subject's center of gravity while the subject attempted to maintain his/her balance.^38^ The equilibrium score quantifies the postural stability of the six sensory conditions. Effective use of individual sensory inputs is determined from the overall pattern of scores on the six conditions. The composite equilibrium score, which is the weighted average of the scores of all sensory conditions, characterizes the overall level of performance. Sensory analysis ratios are used in conjunction with the individual equilibrium scores to identify impairments of individual sensory systems. The somatosensory ratio is the ratio of the balance score of C2 to that of C1. The visual ratio is the ratio of the balance score of C4 to that of C1. The vestibular ratio is the ratio of the balance score of C5 to that of C1. The preference ratio is the ratio of the score of C3 + C6 to that of C2 + C5.^39^

### Procedure

Institutional review and approval from the Ethics Committee was obtained (Number: B.30.2 HAC.0.20.05.04/321). Children in the ADHD and control groups were first evaluated by two child and adolescent psychiatrists by conducting clinical interviews and the K-SADS-PL separately for the children and their parents. All parents gave informed consent for participation, which was consistent with the Code of Ethics of the World Medical Association (Declaration of Helsinki). WISC-R was administered individually on different days, to avoid the possibility of different carryover effects between the ADHD and the control groups, by a clinical psychologist in a quiet room at the pediatric hospital, and the parents filled CPRS-48. Children fulfilling the inclusion and exclusion criteria for the study were further evaluated by performing the experimental balance tasks on another day in the Vestibular Laboratory at the same hospital. A total of 55 children were evaluated, but 9 children were excluded for the following reasons: hearing loss (*n* = 3), Total IQ score below 80 (*n* = 1), learning disorder (*n* = 1), additional handicaps (*n* = 2), aphysiologic pattern in SOT (*n* = 1), and problems in adapting to the test (*n* = 1). Finally, 26 children were included in the ADHD group and 20 children were included in the control group. These 46 children completed all measurements.

### SOT protocol

In this study, a Neurocom SMART Balance Master Dynamic Posturography device was used in SOT. The examiners were three licensed audiologists. The participants stood barefoot on the platform with their feet placed 5.7 cm apart and the medial malleolus aligned with the axis of the platform rotation. Foot position was marked on the platform to ensure consistency between the trials and sessions. The participants wore a harness that was attached overhead and prevented falls but did not limit sway; they were asked to stand quietly with their arms across their chest and their eyes open or closed (depending on the condition). An examiner remained stationed behind each subject for safety throughout the test. Each test condition was completed three times for 20s in the same order. In addition to the standard SOT protocol in which there was no accompanying distractor (silence environment), all protocols were repeated while the participants were listening to relaxing music and white noise through earphones. The MP3 player (Philips Model SA3115/02; Headphones: AY3809) was in a case and placed near the upper chest held by appropriate length strips. Disposable earphone covers were used, buttons were locked during the tests, and the volume level was stabilized to prevent manipulations. The participants were asked to warn the audiologist if the sound was distorted or interrupted. Therefore, SOT was repeated three times for each participant in three different test environments: (1) silence (no background distractor), (2) accompanying background music, and (3) accompanying background noise. Music composed of soothing nature sounds, such as forest and waterfall sounds, was chosen as the music distractor, and “white noise” was chosen as the noise distractor. The order of test environments was selected randomly for every participant, and 5 min breaks were given between the tests and children were encouraged to walk around during the break.

### Statistical analysis

SPSS 18.0 was used for all statistical analyses. Continuous variables were analyzed for normal distribution using the Kolmogorov–Smirnov test with Lilliefors significance correction. Student's *t*-test and the chi-square test were applied to determine the differences in continuous and categorical variables between the two groups, respectively. Effect size values, computed using the d statistics, were also used to reflect the difference between two means.^40^ Repeated measures analysis of variance was applied with Bonferroni adjustment in pairwise comparisons to compare balance performances in three different test environments in the groups. For sensory analysis ratios, the Mann–Whitney *U*-test was used to compare balance performance between the groups, and Friedman's two-way test was used to reveal differences between the groups. Significant level was accepted as 0.05.

## Results

By design gender and age were not significantly different in the ADHD and the control groups. In addition, height and total IQ scores were not different between the two groups. Children in the ADHD group had lower scores on General Information, Similarities, Arithmetic, Judgment, Digit Span and Digit Symbol subscales of the WISC–R but the differences could not reach statistical significance (*p* = 0.124, *p* = 0.362, *p* = 0.216, *p* = 0.193, *p* = 0.095, *p* = 0.144). As expected children in the ADHD group had significantly higher scores on the CPRS-48 (Table 1).

**Table 1:** Main characteristics of the ADHD and the control groups.

	ADHD (*n* = 26)	Control (*n* = 20)	Statistics	*p*-Value
Age (months)	109.50 ± 21.38	116.45 ± 15.02	1.236	0.223
Male/female	20/6	14/6	0.281	0.596
Height (cm)	136.76 ± 10.21	138.90 ± 12.77	0.625	0.535
WISC-R total IQ	104.68 ± 12.67	102.75 ± 11.17	−0.535	0.596
WISC-R verbal IQ	99.24 ± 14.11	102.05 ± 11.08	0.728	0.470
WISC-R performance IQ	109.44 ± 13.93	103.10 ± 12.68	−1.578	0.122
CPRS-48 scores	21.89 ± 10.54	10.82 ± 7.26	−3.624	**0.001**^a^

ADHD, attention deficit hyperactivity disorder; WISC-R, Wechsler Intelligence Scale for Children-Revised; CPRS-48, The Conners’ Parent Rating Scale.

a indicates *p*<0.05.

**Figure 1:**
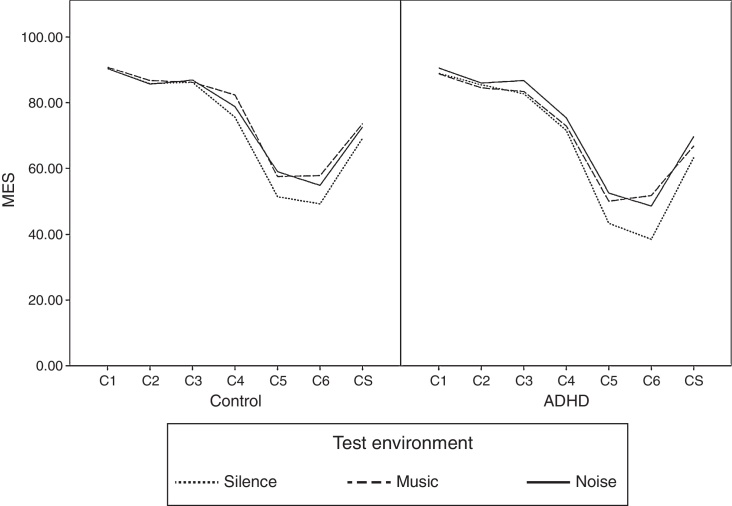
Mean equilibrium scores of the control and the ADHD groups in the three test environments. MES, mean equilibrium scores; ADHD, Attention Deficit Hyperactivity Disorder; C1, eyes open, stable platform; C2, eyes closed, stable platform; C3, visual disorientation (sway referenced vision), stable platform; C4, eyes open, unstable platform; C5, eyes closed, unstable platform; and C6, visual disorientation, unstable platform; CS, composite score.

Mean Equilibrium Scores (MES) of the control and the ADHD groups in the three test environments, namely silence, relaxing music and white noise are shown in Fig. 1. Auditory distractors affected the Composite Scores (CS) positively for both groups. C4, C5 and C6 were the conditions more affected in both of the groups. When the changes are tested for statistical significance, MES did not change significantly for C1, C2, C3, C4 and C5 in the three test environments in the ADHD group. However, children in the ADHD group showed significantly different balance performances for C6 and the CS in the three test environments (*p* = 0.015, *p* = 0.009). For C6 children in the ADHD group showed better balance performance in the listening to music and the white noise environment than in the silence environment (*p* = 0.042, *p* = 0.006). MES in the music and the noise environment were not significantly different (*p* = 0.990). For CS children in the ADHD group showed better balance performance in the white noise environment than in the silence environment (*p* = 0.001). MES in the music and the noise environment were not significantly different (*p* = 0.438). In the control group balance performance did not significantly change for C1, C2, C3, C5, C6 and CS in the three test environments. Unlike the ADHD group, children in the control group showed significantly different balance performances for C4 in the three test environments (*p* = 0.032). For C4, children in the control group showed significantly better performance in the music environment than in the silence environment (*p* = 0.012). MES in the silence and the noise and music and the noise environment were not significantly different (*p* = 0.829, *p* = 0.208).

**Figure 2:**
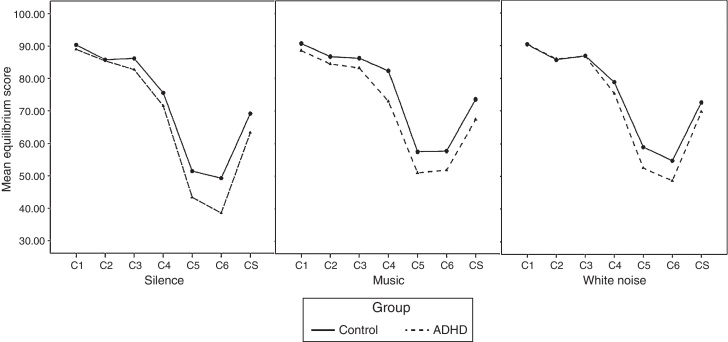
Comparison of the mean equilibrium scores of the ADHD and the control groups in the three test environments. ADHD, attention deficit hyperactivity disorder; C1, eyes open, stable platform; C2, eyes closed, stable platform; C3, visual disorientation (sway referenced vision), stable platform; C4, eyes open, unstable platform; C5, eyes closed, unstable platform; and C6, visual disorientation, unstable platform; CS, composite score.

Table 2 presents MES for all the SOT conditions and the CS for the both groups in the three test environments. All MES for each SOT condition in all the environments were lower in the ADHD group. However only MES for C4 and the CS in the music environment reached statistical significance with small effect sizes (Table 2). The overall balance performances of the ADHD and the control groups in each environment are presented in Fig. 2. The MES for all the SOT conditions and CS were higher in the control group, but the overall balance performances of the two groups in each environment were not significantly different (*p* = 0.429, *p* = 0.084, *p* = 0.833).

**Table 2:** Mean equilibrium scores in the ADHD and the control groups in the three test environments.

SOT condition	ADHD (mean ± SD)	Control (mean ± SD)	Statistics	*p*-Value	*d*
*Silence*					
C1	88.96 ± 5.63	90.35 ± 3.29	1.050	0.300	0.008
C2	85.43 ± 6.17	85.79 ± 3.18	0.253	0.802	0.001
C3	82.71 ± 9.81	86.17 ± 3.61	1.653	0.108	0.290
C4	71.53 ± 15.64	75.54 ± 9.84	1.063	0.294	0.004
C5	43.36 ± 17.04	51.47 ± 15.50	1.682	0.100	0.360
C6	38.52 ± 20.46	49.29 ± 21.22	1.732	0.091	0.440
Composite score	63.30 ± 12.42	69.15 ± 8.54	1.886	0.066	0.042
*Music*					
C1	88.75 ± 6.26	90.76 ± 3.40	1.388	0.173	0.041
C2	84.53 ± 9.07	86.72 ± 4.90	1.049	0.300	0.022
C3	83.39 ± 9.92	86.22 ± 4.72	1.277	0.209	0.025
C4	72.88 ± 13.28	82.34 ± 6.67	3.149	**0.003**^a^	0.150
C5	50.12 ± 20.17	57.52 ± 16.34	1.373	0.177	0.020
C6	51.84 ± 17.85	57.77 ± 19.92	1.039	0.305	0.019
Composite score	66.80 ± 10.69	73.55 ± 8.70	2.294	**0.027**^a^	0.080
*Noise*					
C1	90.54 ± 3.56	90.39 ± 3.23	−0.150	0.881	0.001
C2	85.96 ± 5.38	85.64 ± 3.85	−0.235	0.815	0.001
C3	86.72 ± 4.77	86.87 ± 3.96	0.118	0.907	0.002
C4	75.36 ± 12.88	78.78 ± 9.94	1.015	0.316	0.010
C5	52.56 ± 17.36	58.98 ± 15.60	1.318	0.195	0.022
C6	48.64 ± 21.91	54.83 ± 19.55	1.009	0.319	0.009
Composite score	69.69 ± 10.54	72.60 ± 9.08	1.003	0.322	0.009

SOT, Sensory Organization Test; ADHD, attention deficit hyperactivity disorder; Effect size *d*, mean of ADHD group-mean of control group/pooled standard deviation of two groups.

a indicates *p*<0.05.

The sensory analysis test results showed that children in the ADHD and the control group had similar somatosensory and preference ratios in the three test environments. Children in the ADHD group had lower visual and vestibular ratios but the difference reached significance only for the visual ratio in the music environment (Table 3). Within the ADHD group the somatosensory, visual, vestibular and preference ratios did not significantly differ in the three test environments (*p* = 0.808, *p* = 0.071, *p* = 0.254, *p* = 0.302). Whereas, within the control group the visual ratios changed significantly between the three test environments (*p* = 0.014). The visual ratio in the silence environment was significantly lower then only the music environment in the control group (*p* = 0.034, *p* = 0.144). The somatosensory, vestibular and preference ratios did not significantly differ in the three test environments in the control group (*p* = 0.294, *p* = 0.099, *p* = 0.709).

**Table 3:** Sensory analysis ratios in the ADHD and the control groups in the three test environments.

Sensory analysis ratios	ADHD (mean ± SD)	Control (mean ± SD)	Statistics	*p*-Value
*Silence*				
Somatosensory	0.96 ± 0.04	0.95 ± 0.04	282.50	0.608
Visual	0.84 ± 0.19	0.86 ± 0.10	202.50	0.199
Vestibular	0.53 ± 0.30	0.64 ± 0.18	195.50	0.152
Preference	0.94 ± 0.12	0.96 ± 0.14	226.50	0.449
*Music*				
Somatosensory	0.95 ± 0.04	0.97 ± 0.02	222.00	0.383
Visual	0.84 ± 0.16	0.95 ± 0.07	123.50	**0.002**^a^
Vestibular	0.65 ± 0.24	0.71 ± 0.20	201.00	0.190
Preference	0.98 ± 0.11	1.00 ± 0.05	233.00	0.518
*Noise*				
Somatosensory	0.96 ± 0.04	0.95 ± 0.04	285.50	0.561
Visual	0.89 ± 0.19	0.94 ± 0.10	213.00	0.292
Vestibular	0.63 ± 0.26	0.72 ± 0.17	212.50	0.291
Preference	0.96 ± 0.07	0.97 ± 0.08	248.00	0.782

ADHD, attention deficit hyperactivity disorder.

a indicates *p*<0.05.

## Discussion

The main purpose of the current study was to investigate the effects of distractors on upright balance performance in children with ADHD. When there were no distractors, the balance scores under all SOT conditions and the composite equilibrium score were lower in the ADHD group. Although the differences were not statistically significant, the decreases in the scores were more apparent in C4, C5, C6, and the composite score. These findings indicate that children with ADHD had worse upright balance performance when they had to rely on visual and vestibular signals instead of somatosensorial signals. Shum et al. found worse balance scores under all SOT conditions except in C1 and lower somatosensorial, vestibular, and visual ratios in children with ADHD.^28^ Their study design was similar to that of ours; however, they included a higher number of ADHD children (*n* = 43) and calculated balance performance after correcting for the physical activity level. They argued that somatosensorial information in addition to visual and vestibular signals affects balance control. The lower number of participants in our study, and the correction of balance scores according to physical activity level in the previous study may explain why the differences were not statistically significant in our study. In another study, Buderath et al. found increased sway area in C4 in children with ADHD (*n* = 10), and an increased number of falls in C5 and C6 in children with chronic cerebellar lesions (*n* = 7).^26^ The abnormalities in dynamic SOT were more apparent in C4, C5, and C6; these results were consistent with those of our study. It is known that vestibular and visual signals are crucial in maintaining postural control. Several brain magnetic resonance imaging studies have shown anatomical abnormalities in the cerebellar vermis, which is important in postural and gait control.^14^, ^16^, ^22^ In addition to the cerebellum, fronto-striatal-cerebellar system dysfunction was observed in children with ADHD.^12^, ^13^, ^41^ In addition to lower balance scores in C4, C5, and C6, particularly the visual and vestibular ratios were lower in sensory analysis in the ADHD group in the silence environment. Although not statistically significant (*p* = 0.199, *p* = 0.152), this result may be clinically important. Thus, it can be said that in the silence environment, the ADHD patients’ abilities to use input from the visual and the vestibular systems are affected.

It is known that quiet standing is not a pure reflex mechanism and is strongly associated with cognitive processes. In daily life, static or dynamic balance control is performed simultaneously with cognitive tasks, such as listening to music while running.^42^ The attentional needs for balance control vary depending on the postural task, age of the individual, and their balance abilities.^25^ To investigate the role of cognition in postural control, dual-task studies have been conducted.^21^, ^43^, ^44^ In general, it has been thought that a second task may adversely affect balance control.^45^ Shorer et al. included 24 children with ADHD and measured sway velocity under single-task and auditory–memory attention-demanding dual-task conditions.^23^ In contrast to their hypothesis, concurrent auditory–memory cognitive tasks did not have a negative effect on postural control in either the ADHD group or the control group. Jacobi-Polishook et al. investigated the effect of methylphenidate on postural stability under single- and dual-task conditions (*n* = 24), with listening to music as one of the tasks. Methylphenidate administration resulted in better postural stability performance under the dual-task condition and while listening to music.^18^ The researchers concluded that enhanced attention abilities improved balance performance. In Söderlund et al.’s study, although noise was perceived as adversely affecting cognitive performance, white noise had a positive effect on cognitive performance in children with ADHD. The researchers explained this result by a possible underlying mechanism, termed moderate brain arousal model, which supposes that noise in the environment causes internal noise in the neural system through the perceptual system and that this noise affects the neurotransmitter systems and improves cognitive performance.^32^ The possible enhancing effects of distractors on cognitive process have been discussed in several other reports.^31^, ^46^, ^47^, ^48^ They hypothesized that arousal, activation, and effort modulate children's information-processing abilities and distracting information can enhance performance temporarily, possibly by increasing arousal to an optimal level.^48^ Similarly our results showed that, even though not reaching statistical significance levels, auditory distractors affected the general balance performance positively for both groups. More challenging conditions, using an unstable platform (C4, C5, C6) with distorted somatosensory signals, were more affected by auditory distractors in both groups. It can be assumed that more challenging conditions need more cognitive effort and more information-processing, therefore more affected by the possible enhancing effects of auditory distractors. Based on the above summarized knowledge and our congruent results it can be hypothesized that auditory distraction such as music and white noise is not always distracting in children with ADHD and can also have beneficial effects. The positive effect of music and white noise reached significance level in C6 in the ADHD group. C6 is assumed to be the most challenging condition with conflicting somatosensory and visual signals and leaving only vestibular signals available. In the ADHD group the positive effects of the auditory distractors became more apparent and significant as the condition got harder. In the control group, the positive effect reached significance in only C4 while listening to relaxing music only. Extending the previous hypothesis, we think that in terms of balance control, children with ADHD benefit more than typically developing children from auditory distractors in challenging conditions needing more cognitive processes. In a way, the positive effects of auditory distractors in typically developing children and their ADHD counterparts can be compared to those of stimulants. It is known that stimulants enhance cognitive functions in healthy individuals too but the enhancing effects are much more significant in people with ADHD.^49^ In the sensory ratio analysis children in the ADHD group had lower visual and vestibular ratios but the difference reached significance only for the visual ratio in the music environment. Deficits in visual processing and integration have been previously reported in children with ADHD.^9^, ^50^ Similarly Shum et al. showed the greatest between group differences in the visual ratio. In addition, in our study when the ADHD and the control groups were compared MES for C4 and the CS reached statistical significance.^28^ In fact C4 is the condition where the children are forced to rely more on the visual information when somatosensory signals are disrupted. We think it can be speculated that the visual system may be the most involved system in contributing to the balance deficits among children with ADHD.

Strengths of this current study include a DSM-IV-based, multi-method (interview and rating scale) diagnostic procedure for ADHD, matching of the children in the ADHD group in terms of age and gender. There are also several limitations with the study. We did not have the opportunity to administer the diagnosis according to the DSM-V but the period in which the study was carried out DSM-IV-TR was the most reliable method congruent with a semi-structured interview for performing the assessment. Even though the groups were matched for age and gender, there may be other variations that cannot be controlled influencing the children's performance. Due to small sample size the number of significant findings may be somewhat reduced. More importantly, the enhancing effect of white noise and relaxing music may change with signal amplitude.^32^ Therefore, different signal amplitudes should have been tested. In addition, we did not investigate the effect of background speech signals as a distractor; examining this effect may provide more realistic information similar to that in a classroom setting. Moreover, age range may influence real balance performance; thus, maturation of somatosensorial information completes by the age of 3–4 years, whereas maturation of visual and vestibular information in balance is still developing at 15 years of age.^38^ These characteristics may explain the finding of better scores in C1, C2 and C3 and worse scores in C4, C5 and C6. However, the results obtained in the vestibular and visual ratio analysis possibly reflect the real capability inefficiency of vestibular and visual signals in children with ADHD.

## Conclusion

To the best of our knowledge, this is the first study evaluating balance performance in children with ADHD under the effects of auditory distractors. Although more studies are needed, our results indicate that distractors may have enhancing effects on upright balance performance and it may be advantageous to investigate the effects of early vestibular rehabilitation in this population.

## Conflicts of interest

The authors declare no conflicts of interest.
